# Transplante Cardiopulmonar: Quando Indicar?

**DOI:** 10.36660/abc.20200536

**Published:** 2021-02-19

**Authors:** Paulo Manuel Pêgo Fernandes, Gabriela Favaro Faria, Flávio Pola dos Reis, Luís Gustavo Abdalla, José Eduardo Afonso, Fernando Bacal

**Affiliations:** 1 Hospital Israelita Albert EinsteinSão PauloSPBrasilHospital Israelita Albert Einstein, São Paulo, SP - Brasil; 2 Hospital das ClínicasFaculdade de MedicinaUniversidade de São PauloSão PauloSPBrasilInstituto do Coração, Hospital das Clínicas HCFMUSP, Faculdade de Medicina, Universidade de São Paulo, São Paulo, SP - Brasil

**Keywords:** Transplante Coração-Pulmão/tendências, Embolia Pulmonar, Hipertensão Pulmonar, Fibrilação Atrial, Litíase, Acidente Vascular Cerebral

## Introdução

O transplante cardiopulmonar (TCP) teve seu auge no final da década de 1980 e início dos anos 1990, com quase 300 transplantes realizados por ano em todo o mundo. Com os avanços no tratamento da insuficiência cardíaca (IC) e pulmonar, esse número caiu consideravelmente, sendo que, em 2017 foram realizados apenas 62 TCPs em todo o mundo.^1,2^ Existe uma enorme discussão sobre o perfil de pacientes que pode se beneficiar do TCP e o melhor momento em que ele deve ser indicado.

## Relato de caso

Relatamos o caso de uma paciente de 46 anos, com diagnóstico de comunicação atrioventricular (CAV), submetida a correção cirúrgica em 2006, que evoluiu com hipertensão pulmonar secundária em pós-operatório tardio. Apresentava antecedentes pessoais como: acidente vascular cerebral em 2014, com hemiplegia à direita, fibrilação atrial crônica e litíase biliar. Referenciada ao serviço de transplante, a paciente foi incluída em lista de espera para o procedimento após discussão multidisciplinar. Evoluiu com necessidade de internação por dispneia e piora da insuficiência cardíaca classe funcional II para IV (de acordo com a New York Heart Association, NYHA). Uma angiotomografia de tórax mostrou tromboembolismo pulmonar crônico (TEPC) das artérias principais e ramos interlobares. Um ecocardiograma transtorácico mostrou disfunção do ventrículo direito (VD) grave com pressão sistólica da artéria pulmonar (PSAP) de 129 mmHg, presença de fluxo bidirecional transeptal, e pelo menos dois orifícios compatíveis com comunicação interatrial (CIA) do tipo *ostium secundum*, medindo 10 mm e 8 mm, respectivamente.

**Figura 1:**
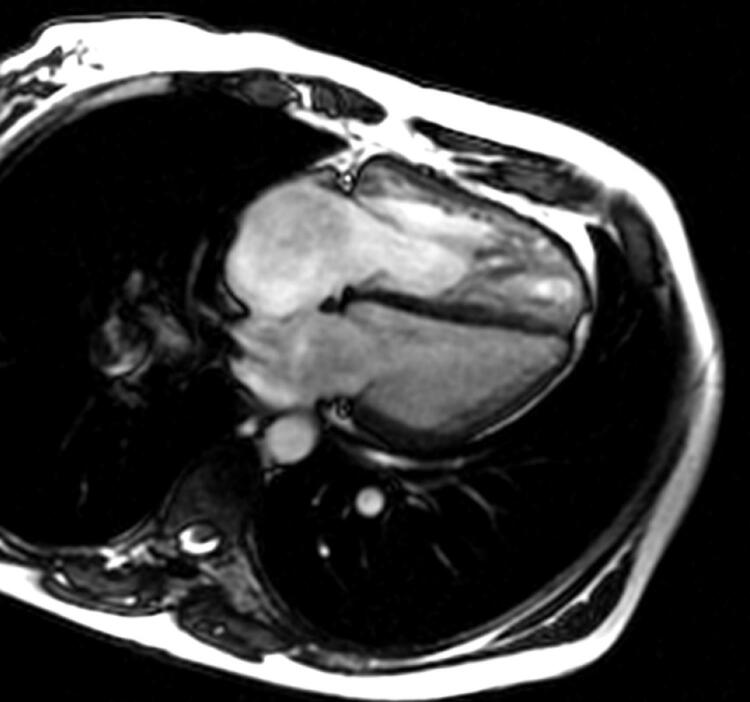
– Ressonância magnética cardíaca mostrando comunicação interatrial.

**Figura 2:**
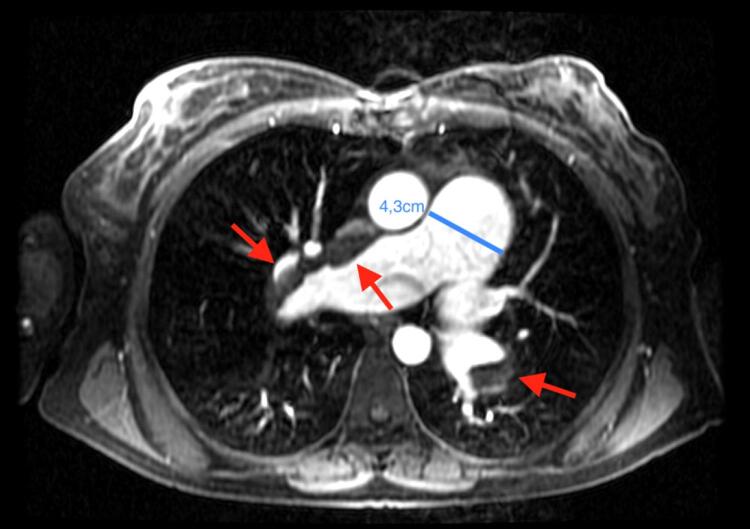
– Ressonância magnética cardíaca evidenciando tromboembolismo na artéria pulmonar direita (seta vermelha) e o aumento do tronco da artéria pulmonar.

A paciente evoluiu com progressão da disfunção cardíaca e piora do quadro clínico (dispneia aos mínimos esforços, cianose central), aumento da pressão sistólica da artéria pulmonar (PSAP = 153mmHg) e necessidade de uso de inotrópico (milrinone) contínuo. Foi realizada uma ressonância magnética cardíaca, que identificou retorno da CIA (Figura 1) e TEPC na artéria pulmonar direita (Figura 2).

Após discussão na Câmara Técnica da Central Estadual de Transplantes, a paciente foi priorizada na fila do transplante e, após cinco meses de internação, o TCP foi realizado no Hospital Israelita Albert Einstein (HIAE). O doador era do sexo masculino, 18 anos, e a causa de morte encefálica tinha sido acidente vascular cerebral hemorrágico (AVCH).

A incisão cirúrgica foi feita por bitoracotomia anterior com esternotomia transversal tipo Clamshell, com instalação da circulação extracorpórea (CEC) por meio da canulação na aorta ascendente e drenagem nas veias cavas superior e inferior. Durante a cardiectomia, os nervos frênicos foram identificados bilateralmente e liberados com margens de segurança.

Durante as pneumonectomias, preservou-se a região do nervo laríngeo recorrente para evitar lesões. O implante do bloco cardiopulmonar iniciou-se pelas anastomoses brônquicas, seguidas da aorta e das veias cavas. O tempo de isquemia do enxerto foi 255 minutos e o tempo de CEC, 195 minutos. Após saída da CEC, a paciente foi submetida a tromboelastograma e coagulograma, corrigido conforme resultado com plaquetas, fibrinogênio e complexo protrombínico, além de dois concentrados de hemácias.

A paciente foi admitida na unidade de terapia intensiva em ventilação mecânica, recebendo 0,5 micro/kg/min de noradrenalina; 0,06 micro/kg/min de vasopressina; 3,7 micro/kg/min de dobutamina. A profilaxia utilizada foi meropenem e vancomicina e, na indução, metilprednisolona e basiliximabe. Para a imunossupressão, utilizamos Tacrolimus, prednisona e micofenolato.

A paciente foi entubada no segundo dia pós-operatório, com relação PO_2_/FiO_2_ de 400. Nas primeiras 72 horas após o transplante, a paciente apresentou disfunção primária do enxerto I (DPE), apenas pela alteração radiológica, sem repercussão clínica. Permaneceu na unidade de terapia intensiva por quatro dias e recebeu alta hospitalar no 34º dia pós-operatório. Atualmente, está em acompanhamento ambulatorial, referindo boa qualidade de vida.

## Discussão

A International Society for Heart and Lung Transplantation (ISHLT) reporta que a maior indicação para o TCP continua sendo a hipertensão pulmonar, devido à hipertensão arterial pulmonar idiopática ou secundária a doenças cardíacas congênita (como a Sindrome de Eisenmenger), que representa 60% a 70% dos transplantes nas últimas três décadas, seguida da Fibrose Cística com 14,9%.^3,4^

A opção pelo transplante isolado de coração e pulmão para os pacientes que antigamente seriam tratados com o TCP, bem como os avanços no tratamento da hipertensão pulmonar refletiu na diminuição do número de TCP realizados.

Os exames pré-operatórios devem ser criteriosamente analisados, pois os pacientes que serão indicados e submetidos ao TCP podem ter comprometimento de outros órgãos, como fígado e rim, além de congestão venosa sistêmica crônica. Outro ponto a ser verificado é se o receptor tem painel imunológico positivo, pois, devido a procedimentos prévios com transfusões, o paciente pode ser sensibilizado.^5^

O manejo pós-operatório do TCP é semelhante ao dos pacientes submetidos ao transplante pulmonar isoladamente. As causas comuns de morte nos primeiros 30 dias são falência do enxerto, complicações técnicas e infecção. A síndrome de bronquiolite obliterante (BOS) e a disfunção do aloenxerto pulmonar (DEP) continuam sendo as principais causas de mortalidade no primeiro ano.^6^

Há na literatura internacional uma grande discussão sobre a necessidade e a indicação do TCP ou quando recomendar o transplante pulmonar ou cardíaco isoladamente. No Brasil, a 3ª Diretriz Brasileira de Transplante Cardíaco aconselha que, em casos de hipertensão pulmonar fixa, o TCP pode ser considerado.^7^ Porém, alguns pontos devem ser levados em consideração: a anatomia, o agravamento da insuficiência ventricular, hipertensão, condições clínicas e hemodinâmicas do paciente e piora da qualidade de vida, índice cardíaco e disfunção renal.^8^

Uma desvantagem do TCP é o uso de um doador para um paciente, enquanto poderiam ser realizados um transplante cardíaco e pulmonar bilateral ou dois unilaterais, em dois ou três pacientes.^9^

Existem muitos pacientes no Brasil que podem se beneficiar do TCP, seja por cardiopatia congênita ou por Hipertensão Arterial Pulmonar (HAP) de qualquer etiologia, em que o acometimento é severo e irreversível.^10,11^

## Conclusão

O TCP deve ser considerado como opção terapêutica para pacientes cuidadosamente selecionados. O momento de indicação para o transplante, antes que a doença se agrave, é de fundamental importância para atingir bons resultados. O manejo desses pacientes requer cuidado complexo e multidisciplinar. Há uma demanda em parte oculta em nossa população que pode se beneficiar desse tipo de transplante.
