# Supplementary material for: Transplante Cardiopulmonar: Quando Indicar?
Source: Arq Bras Cardiol. 2021 Feb 19;116(2):346–8. [Article in Portuguese] doi: 10.36660/abc.20200536 (PMC7909965; doi:10.36660/abc.20200536)
Supplement: Translation

# Translation: Cardiopulmonary Transplantation: When to Indicate?

**Authors:** Paulo Manuel Pêgo Fernandes, Gabriela Favaro Faria, Flávio Pola dos Reis, Luís Gustavo Abdalla, José Eduardo Afonso, Fernando Bacal

**Affiliations:** 1 BrazilHospital Israelita Albert Einstein, São Paulo, SP - Brazil; 2 BrazilInstituto do Coração, Hospital das Clínicas HCFMUSP, Faculdade de Medicina, Universidade de São Paulo, São Paulo, SP - Brazil

**Keywords:** Heart–Lung Transplantation/trends, Pulmonary Embolism, Hypertenson Pulmonary, Atrial Fibrillation, Lithiasis, Stroke

## Introduction

Cardiopulmonary transplantation (CPT) peaked in the late 1980s and early 1990s, with almost 300 transplants performed each year worldwide. With the advances in the treatment of heart and lung failure, this number has dropped considerably, and in 2017 only 62 CPTs were performed worldwide.^1,2^ There is a huge discussion about the profile of patients who can benefit from CPT and the best time for its indication.

## Case report

We describe the case of a 46-year-old female patient diagnosed with atrioventricular communication (AVC), who underwent surgical correction in 2006 and developed secondary pulmonary hypertension in the late postoperative period. Her personal history was: stroke in 2014, with right hemiplegia, chronic atrial fibrillation, and biliary lithiasis. Referred to the transplant service, the patient was put on a waiting list for the procedure after a multidisciplinary discussion. She evolved with need for hospitalization due to dyspnea and worsening of heart failure, from functional class II to IV (according to the New York Heart Association, NYHA). A chest computed tomography (CT) scan showed chronic pulmonary thromboembolism (PTE) of the main arteries and interlobar branches. A transthoracic echocardiogram showed severe right ventricular (RV) dysfunction with pulmonary artery systolic pressure (PASP) of 129 mmHg, presence of transeptal bidirectional flow, and at least two orifices compatible with *ostium secundum* type atrial septal defect, measuring 10 mm and 8 mm, respectively.

The patient evolved with progression of cardiac dysfunction and worsening of clinical picture (dyspnea on minimal exertion, central cyanosis), increased pulmonary artery systolic pressure (PASP = 153mmHg) and need for continuous inotropic use (milrinone). A cardiac magnetic resonance imaging (MRI) was performed and identified return of the AVC (Figure 1) and PTE in the right pulmonary artery (Figure 2).

**Figure 1:** – Cardiac magnetic resonance imaging showing interatrial communication.

**Figure 2:** – Cardiac magnetic resonance imaging showing thromboembolism in the right pulmonary artery (red arrow) and an enlarged pulmonary artery trunk.

After a discussion at the Technical Chamber of the State Transplant Center, the patient was prioritized in the transplant wait line and, after five months of hospitalization, the CPT was performed at Hospital Israelita Albert Einstein (HIAE). The donor was an 18-year-old male, whose cause of brain death had been hemorrhagic stroke.

The surgical incision was made by anterior thoracotomy with Clamshell-type transverse sternotomy, and the installation of extracorporeal circulation (ECC) through cannulation in the ascending aorta and drainage in the superior and inferior vena cava. During cardiectomy, phrenic nerves were identified bilaterally and released with safety margins.

During pneumonectomy, the region of the recurrent laryngeal nerve was preserved to prevent injuries. The implantation of the cardiopulmonary block started with bronchial anastomoses, followed by the aorta and vena cava. The graft ischemia time was 255 minutes and the ECC time was 195 minutes. After being removed from ECC, the patient was submitted to a thromboelastogram (TEG) and a coagulogram, corrected according to result with platelets, fibrinogen and prothrombin complex, in addition to two red blood cell concentrates.

The patient was admitted to the intensive care unit (ICU) in mechanical ventilation, receiving 0.5 micro/kg/min of norepinephrine; 0.06 micro/kg/min of vasopressin; 3.7 micro/kg/min of dobutamine. The prophylaxis used was meropenem and vancomycin and, in induction, methylprednisolone and basiliximab. For immunosuppression, we used Tacrolimus, prednisone and mycophenolate.

The patient was extubated on the second postoperative day, with a PO_2_/FiO_2_ ratio of 400. In the first 72 hours after transplantation, the patient had primary graft dysfunction I (PGD), only due to a radiological alteration without clinical repercussion. She remained in the ICU for four days and was discharged on the 34^th^ postoperative day. Currently, she is under outpatient follow-up, reporting a good quality of life.

## Discussion

The International Society for Heart and Lung Transplantation (ISHLT) reports that the major indication for CPT is pulmonary hypertension, due to idiopathic pulmonary arterial hypertension or secondary to congenital heart disease (such as Eisenmenger Syndrome), which represents 60% a 70% of transplants in the past three decades, followed by Cystic Fibrosis, with 14.9%.^3,4^

The option for isolated heart and lung transplantation for patients who would previously be treated with CPT, as well as advances in the treatment of pulmonary hypertension, are reflected in the decrease in number of CPTs performed.

Preoperative exams must be carefully analyzed, as patients who will be referred and submitted to CPT may have involvement of other organs, such as liver and kidney, in addition to chronic systemic venous congestion. Another point to be verified is whether the recipient has a positive immune panel, because previous transfusion procedures could sensitize the patient.^5^

The postoperative management of CPT is similar to that of patients undergoing lung transplantation alone. Common causes of death in the first 30 days are graft failure, technical complications, and infection. Bronchiolitis obliterans syndrome (BOS) and pulmonary allograft dysfunction remain the main causes of mortality in the first year.^6^

There is a discussion in the international literature about the need and indication for CPT or when to recommend lung or heart transplantation alone. In Brazil, the 3rd Brazilian Guidelines on Cardiac Transplantation advises that, in cases of fixed pulmonary hypertension, CPT can be considered.^7^ However, some points must be taken into account: anatomy, worsening of ventricular failure, hypertension, clinical conditions and hemodynamics of the patient, worsened quality of life, cardiac index and renal dysfunction.^8^

A disadvantage of CPT is the use of one donor for a patient, while a bilateral or two unilateral heart and lung transplantation could be performed in two or three patients.^9^

There are many patients in Brazil who can benefit from CPT, whether due to congenital heart disease or Pulmonary Arterial Hypertension (PAH) of any etiology, in which the involvement is severe and irreversible.^10,11^

## Conclusion

CPT should be considered as a therapeutic option for carefully selected patients. The time for referral for transplantation, before the disease gets worse, is of utmost importance to achieve good results. The management of these patients requires complex and multidisciplinary care. There is a hidden demand in our population that can benefit from this type of transplantation.
